# Enhancing the thermoelectric properties of Sr_1−*x*_Pr_2*x*/3_□*_x_*_/3_TiO_3±*δ*_ through control of crystal structure and microstructure

**DOI:** 10.1098/rsta.2019.0037

**Published:** 2019-07-08

**Authors:** Dursun Ekren, Feridoon Azough, Robert Freer

**Affiliations:** School of Materials, University of Manchester, Manchester M13 9PL, UK

**Keywords:** perovskite, thermoelectric oxide, ordering, phonon scattering

## Abstract

A-site deficient perovskites are among the most important *n*-type thermoelectric oxides. Ceramics of Sr_1−*x*_Pr_2*x*/3_□*_x_*_/3_TiO_3_ (*x* = 0.1–1.0) were prepared by solid-state reaction at 1700–1723 K using highly reducing atmospheres. Samples with the highest Sr content had a cubic crystal structure $\left(Pm\bar{3}m\right)$; incorporating Pr with A-site vacancies led to a reduction in symmetry to tetragonal (*I4/mcm*) and then orthorhombic (*Cmmm*) crystal structures. HRTEM showed Pr_2/3_TiO_3_ had a layered structure with alternating fully and partially occupied A-sites and a short-range order along the (100) direction. Electrical conductivity was highest in samples of high symmetry (*x* ≤ 0.40), where the microstructures featured core-shell and domain structures. This enabled a very high power factor of approximately 1.75 × 10^−3^ W m^−1^ K^−2^ at 425 K. By contrast, at high Pr content, structural distortion led to reduced electron transport; enhanced phonon scattering (from mass contrast, local strain and cation–vacancy ordering) led to reduced, glass-like, thermal conductivity. Carbon burial sintering increased the oxygen deficiency leading to increased carrier concentration, a maximum power factor of approximately 1.80 × 10^−3^ W m^−1^ K^−2^ at 350 K and thermoelectric figure of merit of 0.26 at 865 K. The paper demonstrates the importance of controlling both crystal structure and microstructure to enhance thermoelectric performance.

This article is part of a discussion meeting issue ‘Energy materials for a low carbon future’.

## 1. Introduction

Harvesting energy from waste heat using thermoelectricity is attracting increasing interest as a possible sustainable energy technology [1–5]. Thermoelectric materials are often assessed initially by their dimensionless figure of merit, *ZT* = (*σS*^2^/*κ*)*T*; where *σ*, *S* and *κ* are electrical conductivity, Seebeck coefficient and thermal conductivity, respectively. In order to maximize the *ZT*, it is necessary to have materials with high electrical conductivity and Seebeck coefficient, and low thermal conductivity. The interrelationship between these parameters through their dependency on carrier concentration makes the enhancement of *ZT* challenging [1]. Moreover, the low abundance and toxicity of the constituent elements of the traditional thermoelectric materials combined with their restricted operating range limit the large scale application of these materials [2]. By contrast, oxide materials are attractive candidates due to the high abundance and low toxicity of the constituent elements and their high thermal and chemical stability at elevated temperatures [3]. Interest in oxide thermoelectrics was stimulated by the discovery of promising thermoelectric performance for layered NaCo_2_O_4_ [4]; this was followed by other p-type oxides (based on Ca_3_Co_4_O_9_) with encouraging properties [5]. In order to maximize the performance of thermoelectric power generation modules, both p-type and n-type materials with comparable properties are required. However, the performance of n-type thermoelectric oxides is generally below that of the p-type oxides.

SrTiO_3_ is one of the most promising n-type oxide thermoelectric materials, having a thermoelectric power factor, *σS*^2^ comparable to that of the current industry standard Bi_2_Te_3_ [6]. However, the high thermal conductivity of SrTiO_3_ (approx. 10 W m^−1^ K^−1^ at 300 K for a single crystal) resulting from its simple crystal structure limits the overall thermoelectric performance of the material. One of the main challenges for reducing its thermal conductivity is the very small phonon mean-free path, *l*_ph_ which is below 10 nm for the majority of the phonon spectrum [7]. Consequently, atomic-scale defects are necessary to enhance phonon scattering and thereby reduce thermal conductivity. Such defects include substituting cations [8,9], oxygen vacancies [10,11] and lately A-site vacancies [12–14]. Substitution is generally carried out by doping with heavier elements which have different ionic radii than the host, e.g. La in Sr sites and Nb on Ti sites, leading to improved phonon scattering by the formation of a strain field which arises from the distortion of the lattice and the mass difference between host and dopant cations [8]. Oxygen vacancies are also effective point defects for the scattering of phonons [10]. Recently, the effectiveness of A-site vacancies was demonstrated by Popuri *et al.* [12]; they showed that the thermal conductivity of SrTiO_3_ can be dramatically reduced by introducing cation vacancies in Sr_1−*x*_La_2*x*/3_□*_x_*_/3_TiO_3_. Moreover, a glass-like thermal conductivity was obtained with A-site vacancy concentrations greater than 13%. A similar approach was followed by Kovalevsky *et al.* [15], but their work was limited to a low concentration of vacancies. While there have been numerous studies of La doping of SrTiO_3_ [16], there have been comparatively few of other lanthanides. One exception is that of Pr substitution by Dehkordi *et al.* [17]. They prepared Sr_1−*x*_Pr*_x_*TiO_3_ (*x* ≤ 0.15) by Spark Plasma Sintering techniques and achieved a high power factor of approximately 1.68 × 10^−3^ W m^−1^ K^−2^ at 773 K, attributing the enhancement to the formation of Pr-rich grain boundaries. While the *ZT* value is one very useful screening parameter for candidate materials, the Power Factor is in many ways more useful for indicating the potential of the material to generate power in a device [16].

Here, we report a systematic investigation of the structural and thermoelectric properties of A-site deficient Sr_1−*x*_Pr_2*x*/3_□*_x_*_/3_TiO_3_ {*x* = 0.1–1.0} ceramics by examining the effect of Pr/vacancy concentration on the structural and thermoelectric properties. The modification of the crystal structure through cation/vacancy doping is the dominant factor for controlling the thermoelectric properties. We show that carbon burial sintering is an effective and robust method for the preparation of SrTiO_3_-based ceramics, enabling further reduction of the material and enhancement of thermoelectric performance by an increase in carrier concentration.

## 2. Methodology

Samples of Sr_1−*x*_Pr_2*x*/3_□*_x_*_/3_TiO_3_ (*x* = 0.10, 0.25, 0.40, 0.55, 0.70, 0.85, 1.00) were prepared by the conventional mixed oxide route using SrCO_3_ (Sigma Aldrich, greater than 99.9%), TiO_2_ (Sigma Aldrich, greater than 99.9%) and Pr_6_O_11_ (Π-KEM, greater than 99.9%). The Pr_6_O_11_ powders were dried in air at 1173 K for 6 h prior to weighing. The precursor powders were mixed using stoichiometric proportions and calcined at 1523 K for 8 h in air. The resulting powders were formed into disc-shaped pellets using a pressure of 50 MPa and sintered directly at 1700 K in a reducing atmosphere of 5%H_2_–95%Ar for 12 h. To assess the effectiveness of using a graphite bed for producing heavily reduced samples, a second batch of samples was prepared and sintered at 1723 K for 4 h while surrounded by graphite and packed in a closed alumina crucible. The ceramics and corresponding sample codes for the two processing routes are summarized in table 1. Samples will now be identified by these codes.

**Table 1.** Compositions and the corresponding codes for Sr_1−_*_x_*Pr_2*x*/3_□*_x_*_/3_TiO_3_ samples.

		sample code	
compositions	*x*	H_2_–Ar	graphite
Sr_0.90_Pr_0.067_TiO_3±*δ*_	0.10	X10-HA	X10-CM
Sr_0.75_Pr_0.167_TiO_3±*δ*_	0.25	X25-HA	X25-CM
Sr_0.60_Pr_0.267_TiO_3±*δ*_	0.40	X40-HA	X40-CM
Sr_0.45_Pr_0.367_TiO_3±*δ*_	0.55	X55-HA	X55-CM
Sr_0.30_Pr_0.467_TiO_3±*δ*_	0.70	X70-HA	X70-CM
Sr_0.15_Pr_0.567_TiO_3±*δ*_	0.85	X85-HA	X85-CM
Pr_0.67_TiO_3±*δ*_	1.00	X100-HA	X100-CM

XRD and SEM analyses were carried out on polished, bar-shaped samples. X-ray diffraction analysis employed a PANalytical X'Pert Pro X'Celerator Diffractometer with Cu X-ray source (*λ*_Cu−K*α*_ = 1.540598 Å). A continuous scan between 5° and 100° was recorded using 0.0167° step size and a dwell time of 6 s/step. Phase analysis was undertaken with the aid of X'Pert High Score software; Rietveld refinement used TOPAS software [18]. A Philips XL30 FEG-SEM fitted with Bruker EDS detector was employed for microstructure analysis. The average grain size was determined using a linear intercept method [19].

Samples for TEM investigation was prepared by the standard crushing method. The sintered discs were crushed to powder using an agate mortar and pestle. Grains of individual powders were dispersed in chloroform, dropped onto a copper grid covered with a holey carbon film, and then dried. FEI Tecnai G2 TEM with a LaB_6_ source operating at 200 kV and FEI Tecnai TEM with FEG source operating at 300 kV were used for the characterization of the samples. Predominantly, bright field (BF) and selected area electron diffraction (SAED) were used to evaluate structural features in the samples and to determine the crystal structures.

X-ray photoelectron spectroscopy (XPS) data were collected using a Kratos Axis Ultra spectrometer with monochromatic Al K*α* radiation (*E*_source_ = 1486.69 eV). XPS binding energies were calibrated using C 1s peak (284.8 eV). Peak fitting was carried out using the CASA XPS program, allowing the determination of FWHM, peak location and optimal peak shape within the data constraints using a mix of Lorentzian and Gaussian character.

Thermogravimetric analysis (TGA) was undertaken using a Netzsch STA 449 C between 300 and 1700 K. The change in the mass with respect to temperature was recorded during both heating and cooling cycles; the Ti^3+^ concentration and *δ* (oxygen non-stoichiometry) values were determined as described elsewhere [20].

For thermoelectric property measurement, disc-shaped sintered ceramics were cut into bars of 3 × 3 × 12 mm. The electrical conductivity and the Seebeck coefficients were determined simultaneously from 300 to 900 K in a low pressure He atmosphere using ULVAC ZEM-3. Thermal conductivity (*κ*) was obtained from the density (*ρ*), thermal diffusivity (*λ*) and the specific heat capacity (*κ* = *λρ*C_p_) of the samples. Density was determined from mass and dimension measurements, thermal diffusivity was determined in Ar atmosphere using a Netzsch LFA-457 laser flash system, and the heat capacity measured in Ar atmosphere using a Netzsch STA 449 C (TG-DSC).

## 3. Results and discussion

### (a) Bulk properties

The colour of the calcined powders changed from beige to lime green with increasing Pr content in going from *x* = 0.10 to *x* = 1.00 in Sr_1−*x*_Pr_2*x*/3_□*_x_*_/3_TiO_3_. After directly sintering in H_2_–Ar atmosphere, the colour of the samples changed to dark grey, indicating the change in the oxidation state of titanium under reducing conditions [21]. The density of the samples increased with increasing Pr content, but all samples exhibited densities of at least 94% theoretical.

### (b) Phase development

**Figure 1.**
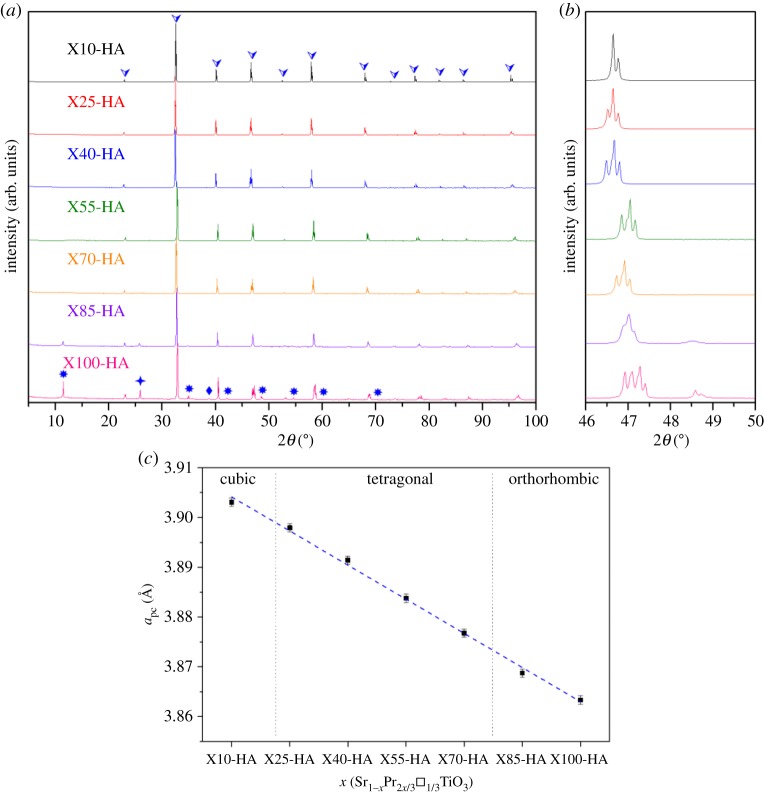
(*a*) XRD spectra of the Sr_1−*x*_Pr_2*x*/3_□*_x_*_/3_TiO_3_ samples prepared in H_2_–Ar atmosphere; (*b*) expanded view of the region near the (200)_p_ peak; (*c*) *a*_pc_ for the samples shown in (*a*): the symbols: 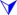, 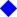, 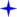, 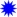 correspond to $Pm\bar{3}m$ SrTiO_3_, R-point reflections, M-point reflections and X-point reflections respectively. (Online version in colour.)

XRD spectra from the samples sintered in H_2_–Ar atmosphere are presented in figure 1*a*. The peaks corresponding to aristotype perovskite phase are present for the whole compositional range. However, additional peaks, corresponding to anti-phase octahedral tilting (R-point reflections), are present in spectra for X25-HA and samples with high values of *x*. The peaks corresponding to cation/vacancy ordering (X-point reflections) and in-phase octahedral tilting of the octahedra (M-point reflections) first become apparent in the X85-HA samples. These differences suggest changes in the crystal structure with increasing Pr and/or vacancy concentration. This can be more clearly seen in the magnified region of the spectra near the (200)_p_ peak (figure 1*b*). It is apparent that sample X10-HA has a cubic structure (*a*^0^*a*^0^*a*^0^) since there is no splitting of the (200)_p_ peak and absence of R-point reflections at 2*θ* ≈ 38°. The second peak observed for this sample is due to Cu K*α*_2_. Increase in the Pr/vacancy concentration with increasing *x* led to the splitting of the (200)_p_ peak and the appearance of R-point reflections for the compositional range of 0.25 ≤ *x* ≤ 0.70. This suggests these samples have a tetragonal crystal structure (*a*^0^*a*^0^*c^–^*). Finally, the appearance of M- and X-point reflections for samples with *x* ≥ 0.85, and the peak splitting of (200)_p_, indicates the samples have an orthorhombic crystal structure (*Cmmm*). These changes in crystal structure with Pr/vacancy concentration are consistent with earlier investigations [13,14,22] of La/vacancy doping of SrTiO_3_. Rietveld refinement of the present XRD spectra confirmed the space groups indicated above. The changes in the pseudo-cubic lattice parameter, *a*_pc_ are presented in figure 1*c*; it can be seen that *a*_pc_ decreased steadily with increasing *x*. This reduction in *a*_pc_ confirms the successful substitution of Sr cations (ionic radius $R_{Sr^{2+}}=1.31\;\text{Å}$) by the much smaller Pr cations ($R_{Pr^{3+}}=1.18\;\text{Å}$) [23].

### (c) Electron microscopy

**Figure 2.**
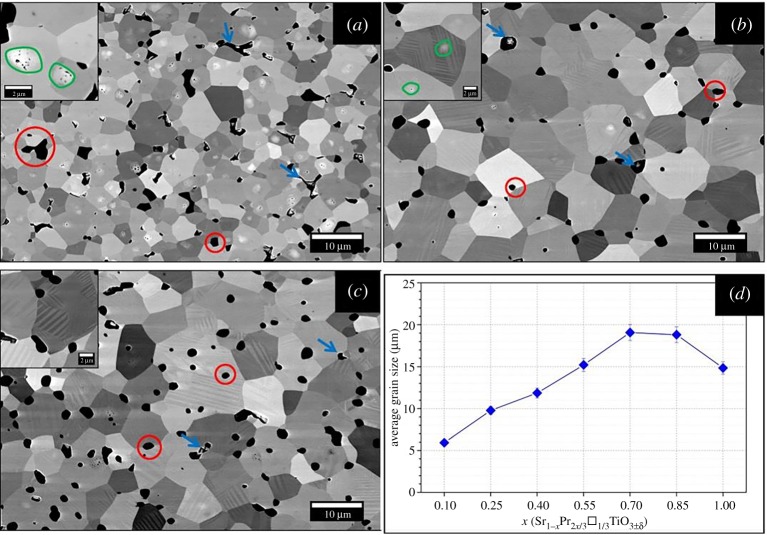
SEM-BSE micrographs showing the microstructure of (*a*) X10-HA, (*b*) X25-HA (*c*) X40-HA samples; features circled and arrowed are identified in the text. (*d*) Average grain size with composition for the samples prepared in H_2_–Ar atmosphere. (Online version in colour.)

The evolution of the microstructure and the change in the average grain size with the composition are shown in figure 2 (backscattered electron, BSE micrographs). Three distinct types of microstructural features are visible; (i) grains with core-shell structures (highlighted by green circles) for samples of *x* ≤ 0.25, (ii) grains with phase transformation-induced domain features for the samples with *x* > 0.25, and (iii) a combination of both for the *x* = 0.25 sample. The average grain size increased from 5.9 µm to 19.1 µm with increasing Pr/vacancy concentration for 0.1 ≤ *x* ≤ 0.70. However, a further increase in *x* led to reduction of average grain size, eventually to 14.9 µm for X100-HA sample. Another important feature visible in the micrographs is the presence of a Ti-rich secondary phase (highlighted by red circles; the arrows indicate porosity). This secondary phase results from the highly reducing conditions used during sample preparation [24] and is often observed in such A-site deficient perovskites; the processes are summarized by equations (3.1) and (3.2) [13,24,25].

3.1 $$A_{1-x}{\mathrm{TiO}}_{3}\overset{\mathrm{Reduction}}{\to }A_{1-x}{\mathrm{TiO}}_{3-\text{δ}}$$

and

3.2 $$A_{1-x}{\mathrm{TiO}}_{3-\delta }\overset{\mathrm{Further}\;\mathrm{Reduction}}{\to }A_{1-x}{\mathrm{TiO}}_{3-\delta -yn}+y\mathrm{Ti}O_{n}.$$

**Figure 3.**
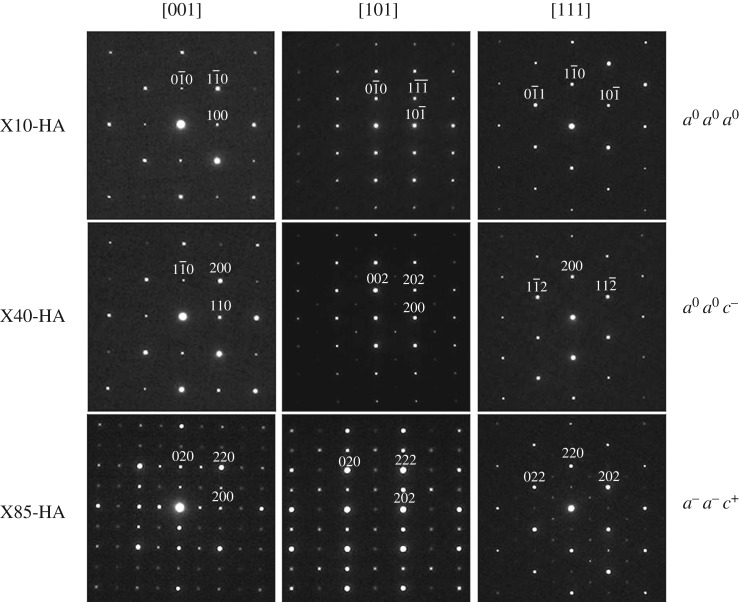
SAED patterns along [001]_p_, [101]_p_ and [111]_p_ zone axes for X10-HA, X40-HA and X85-HA samples. Glazer notations for the compositions are also presented.

Figure 3 shows the SAED patterns along [001]_p_, [101]_p_ and [111]_p_ zone axes for X10-HA, X40-HA and X85-HA samples. No additional spots except for cubic perovskite reflections were observed for X10-HA sample. For 0.25 ≤ *x* ≤ 0.70 samples, no additional reflections were observed along [001]_p_ and [111]_p_ zone axes, while ½{*ooo*} type superlattice reflections were observed along [101]_p_. These latter reflections are linked with out-of-phase tilting of TiO_6_ octahedra and suggest the tilt system for these samples to be *a*^0^*a*^0^*c^–^* according to Glazer's notation [26]. This confirmed the crystal structure to be tetragonal with *I4/mcm* space group, agreeing with XRD analysis. For *x* ≥ 0.85, ½{*ooe*} and ½{*eeo*} type superlattice reflections were observed along [001]_p_ and [101]_p_ zone axes patterns and ½{*ooe*} type reflections were present along [111]_p_ zone axis pattern. The appearance of ½{*ooe*} reflections is linked with the in-phase tilting of TiO_6_ octahedra, while ½{*eeo*} reflections could be associated with anti-parallel cation displacements [27] and long-range cation/vacancy order in A-site [28]. The ½{*ooe*} type reflections along [111]_p_ zone axis only result from in-phase tilting of the octahedra instead of double diffraction of ½{*eeo*}, since ½{*eeo*} type reflections are not permitted for 〈111〉 zone axes according to the Weiss zone law. Thus, the tilt system for these compositions can be assigned as *a^–^a^–^c^+^* using Glazer's notation [26], confirming the crystal structure to be orthorhombic with *Cmmm* space group.

**Figure 4.**
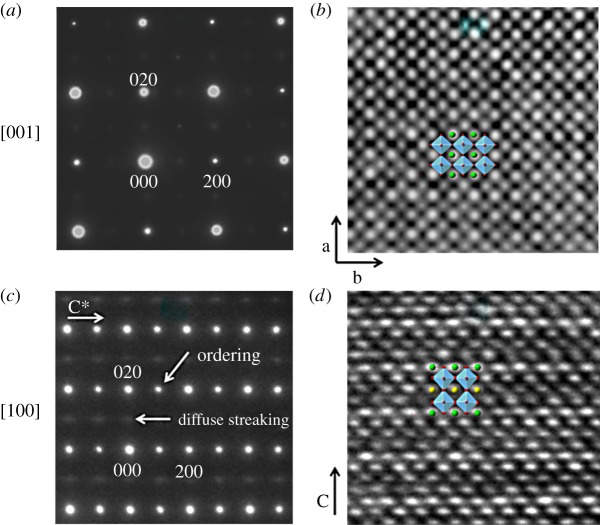
SAED patterns and HRTEM images for X100-HA sample along (*a*,*b*) [001] and (*c*,*d*) [100] zone axes. Projections for the crystal structures along corresponding zone axis are also presented in (*b*,*d*). (Online version in colour.)

HRTEM-SAED data along [001]_p_ and [100]_p_ zone axes for the X100-HA samples (figure 4) can be indexed with *a* ≈ *b* ≈ *c* ≈ *2a*_perovskite_ lattice parameters and *Cmmm* space group. The doubling of the unit cell along the *a*- and *b*-axes is due to the tilting of TiO_6_ octahedra [29]; cation/vacancy ordering leads to the doubling of the *c*-axis. Diffuse streaked ½{*ooe*} type reflections, in addition to the sharp ½{*eeo*} type reflections, were observed in patterns collected along [100]_p_ zone axis. The appearance of such diffuse reflections is explained by the short-range ordering of Pr/vacancies in alternate (001) planes [28]. Structural projections along the corresponding zone axes are overlaid on the HRTEM images in figure 4*b*,*d*.

### (d) Cation valence state and oxygen deficiency

**Figure 5.**
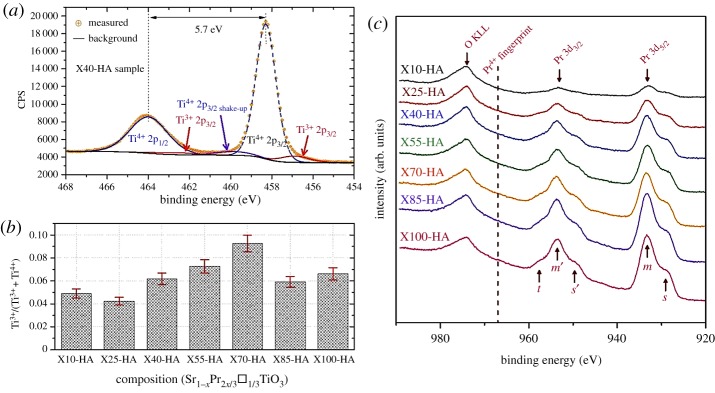
XPS data for the samples prepared in H_2_–Ar atmosphere; (*a*) Example peak fitting for X40-HA sample, (*b*) Ti^3+^ concentration calculated from the fitting process and (*c*) High-resolution Pr 3d spectra for the samples. (Online version in colour.)

Doping SrTiO_3_ with Pr/vacancy using the desired concentrations should not cause any changes in the oxidation state of Ti due to charge neutrality (equation (3.3)), but the formation of oxygen vacancies under reducing conditions leads to the reduction of titanium (equation (3.4)). XPS was initially employed to assess the degree of reduction. Figure 5*a* shows typical peak fitting of Ti 2p for X40-HA sample; Ti 2p_3/2_ and Ti 2p_1/2_ doublets for both 4+ and 3+ valence states are present. Spin-orbital splitting (Δ_s.o._) for doublets was calculated to be approximately 5.7 eV, consistent with earlier reports on SrTiO_3_ [25,30,31]. The concentration of [Ti^3+^] (in terms of Ti^3+^/(Ti^+^+Ti^4+^)) varied with crystal structure and Pr/vacancy concentration between 0.04 and 0.09 (figure 5*b*), suggesting three distinct regions. A decrease in [Ti^3+^] was observed after each structural phase transition with composition but increased with Pr/vacancy concentration within each structural regime. This suggests that for a given crystal structure, it is easier to form oxygen vacancies when increasing Pr/vacancy concentration.

3.3 $$xPr_{2/3}{◻}_{1/3}\mathrm{Ti}O_{3}\overset{\mathrm{SrTi}O_{3}}{\to }\left(\frac{2x}{3}\right)P{r_{\mathrm{Sr}}}^{∙}\;+\left(\frac{x}{3}\right)V_{\mathrm{Sr}}^{\prime \prime }+T{i_{\mathrm{Ti}}}^{x}+{O_{O}}^{x}$$

and

3.4 $$xPr_{2/3}{◻}_{1/3}\mathrm{Ti}O_{3}\overset{\mathrm{SrTi}O_{3-\delta }}{\to }\left(\frac{2x}{3}\right)P{r_{\mathrm{Sr}}}^{∙}+\left(\frac{x}{3}\right)V_{\mathrm{Sr}}^{\prime \prime }+(2\text{δ}){\mathrm{Ti}}_{\mathrm{Ti}}^{\prime }+(1-2\text{δ})T{i_{\mathrm{Ti}}}^{x}+\text{δ}V_{O}{}^{∙∙}.$$

In order to define the valence state of Pr in the samples, Pr 3d core level spectra were also collected (figure 5*c*). The main Pr 3d_5/2_ (m) and Pr 3d_3/2_ (m′) doublets were located at 933.4 and 953.6 eV and spin-orbital splitting, Δ_s.o._ was determined to be approximately 20.2 eV, agreeing with the earlier investigations [32,33]. An additional doublet (*s* and *s*′) at 929.3 and 949.5 eV resulted from the mixing of 3d^9^4f^2^ and 3d^9^4f^3^ configurations [32,34,35]; the peak (*t*) located at 957.6 eV is due to an intra-atomic multiplet effect [34]. These complex features make it very difficult to use any one alone to determine the oxidation state of Pr. However, a 3d4f peak for the final state of PrO_2_, located at 967 eV, is generally used as the ‘fingerprint’ for identification of Pr^4+^ [25,33,34]. Its absence in our samples suggests that Pr is only in the 3+ state, and/or [Pr^4+^] is below the detection limit. In order to determine the degree of oxygen deficiency (*δ*), the sintered samples were oxidized in air and the change in the mass monitored by TGA. It was assumed that the samples were fully oxidized after heat treatment and only Pr^3+^ was present in the samples. The former was confirmed by TGA (no weight change during the cooling cycle; see electronic supplementary material, figure S1a) and the latter deduced by XPS. The *δ* values ranged from 0.05 to 0.12 (detailed in electronic supplementary material, figure S1b as a function of composition). By considering the electroneutrality condition for the nominal composition to be Sr^2+^_1−_*_x_*Pr^3+^_2*x*/3_Ti^3+^_2*δ*_Ti^4+^_1–2*δ*_O^2−^_3−_*_δ_* the [Ti^3+^] content was calculated; values range from 0.11 to 0.24 and are presented in electronic supplementary material, figure S1b. The trends for both *δ* and [Ti^3+^] (electronic supplementary material, figure S1b) are consistent with the compositional dependence of values from XPS data (figure 5*b*), and earlier studies [13,15,36]. However, the values of *δ* and hence [Ti^3+^] from the TGA experiment were higher than those from the XPS analysis. The difference can be explained by the presence of a Ti-rich secondary phase [15,25], observed in SEM micrographs (figure 2) which is likely to be a Magnéli phase, formed to ensure charge compensation.

### (e) Thermoelectric properties

**Figure 6.**
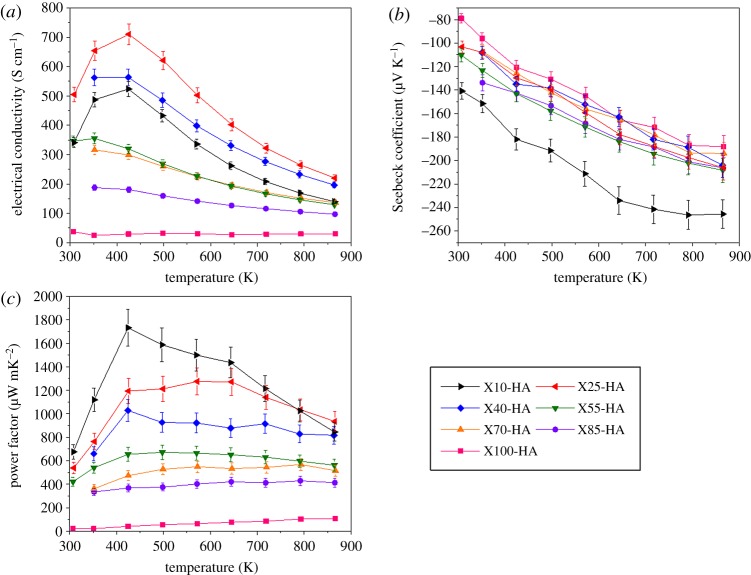
Temperature dependence of (*a*) electrical conductivity, (*b*) Seebeck coefficient and (*c*) thermoelectric power factor for the samples sintered in H_2_–Ar atmosphere. (Online version in colour.)

The temperature dependency of the electrical conductivity of the samples is shown in figure 6*a*; those with low Pr content (i.e. low *x*) exhibited a high and metallic electrical conductivity, similar to that of donor-doped SrTiO_3_ [6,37] while X100-HA had low and semiconductor-like conductivity, similar to that previously reported for Pr_2/3_TiO_3_ [38]. Electrical conductivity initially increased with *x* for *x* ≤ 0.25, while further increase in *x* resulted in the reduction of *σ* over the full temperature range (electronic supplementary material, figure S2a). This is due to the increase in average grain size with increasing Pr/vacancy concentration when *x* ≤ 0.25 (figure 2*d*), while scattering by Pr atoms and vacancies leads to the reduction in *σ* at higher doping levels. Additionally, the variation in Ti^3+^ concentration, and hence the carrier concentration, does not indicate any significant variation with *x* due to the complex defect chemistry observed in the A-site deficient perovskites. Another important observation is the reduction in electrical conductivity of samples with *x* = 1.0 composition, which exhibited both long-range and short-range cation–vacancy ordering, leading to a layered crystal structure with alternating partially and fully occupied A-sites. This could also induce a further reduction of electrical conductivity by the interface scattering of carriers as the typical electron mean-free path is comparable to the interatomic distances [39]. Moreover, the simple cubic crystal structure at low *x* encourages higher *σ* value while the more complex crystal structures developed in samples with high Pr content resulted in low *σ* values [40]. Finally, the Ti-based, Magnéli phases in the samples could be beneficial in this context as they are known to have high electrical conductivity [41,42].

The n-type nature of the samples is apparent from the temperature dependence of the Seebeck coefficients (figure 6*b*). The absolute value of the Seebeck coefficient, |S| decreased with increasing *x* as expected (electronic supplementary material, figure S2b); it is well established that SrTiO_3_ has a high |S| because of its high carrier effective mass, m* [43]. Furthermore, the reduction of the unit cell volume with *x* (figure 2*d*) causes increased overlap of Ti 3d orbitals [15,40,44] and also reduction of the local symmetry of TiO_6_ octahedra. As a result, m* decreases and |S| reduces [45]. Interestingly, the reduction in the symmetry of TiO_6_ due to the change from cubic to tetragonal structure has a more significant effect on |S| than the transition from tetragonal to orthorhombic structure. Moreover, it can also be seen from electronic supplementary material, figure S2b that for 0.25 ≤ *x* ≤ 1.0 the variation in the Seebeck coefficients is limited, indicating that the Pr/vacancy concentration does not directly affect the band structure since the band gap is primarily controlled by O 2p and Ti 3d orbitals rather than the distortion of TiO_6_ octahedra as explained above.

The maximum thermoelectric power factor for the samples, *σ*S^2^, was approximately 1.75 × 10^−3^ W m^−1^ K^−2^ at 425 K and achieved with *x* = 0.1 (figure 6*c*). However, the power factor declined with increasing Pr content and the *x* = 1.0 sample exhibited a maximum of approximately 0.11 × 10^−3^ W m^−1^ K^−2^ at 865 K. An important observation is the relatively high average *σα*^2^ value, exceeding 0.9 × 10^−3^ W m^−1^ K^−2^, over a wide temperature range (425 K < *T* < 875 K) for the samples with *x* ≤ 0.40. Such behaviour is desired for practical applications since the power output of a thermoelectric device is proportional to the power factor [46]. The values obtained in this study are comparable with, and in some cases higher than, the well-established SrTiO_3_-La_2/3_TiO_3_ solid solutions for which a maximum value of approximately 1.6 × 10^−3^ W m^−1^ K^−2^ at 475 K was reported [14]. However, high power factor values, (decreasing from approximately 2.46 to 1.68 × 10^−3^ W m^−1^ K^−2^ for 323–773 K) were reported by Dehkordi *et al.* [17] for Pr-doped (less than or equal to 12.5%) SrTiO_3_ prepared by SPS, while much lower values (maximum of approximately 1.35 × 10^−3^ W m^−1^ K^−2^ at 525 K) achieved by Kovalevsky *et al.* [9] using conventional sintering methods.

**Figure 7.**
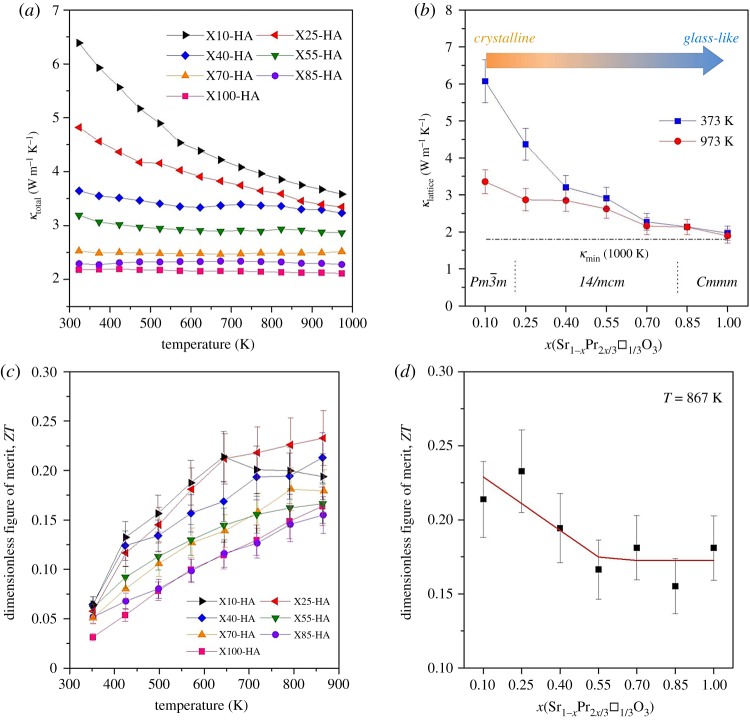
(*a*) Temperature dependence of total thermal conductivity, (*b*) lattice component of thermal conductivity at 373 and 973 K as a function of composition, (*c*) variation in the dimensionless figure of merit, *ZT* with temperature, and (*d*) the *ZT* values at 867 K as a function of composition for the samples prepared in H_2_–Ar atmosphere. (Online version in colour.)

Total thermal conductivity (*κ*_total_) varied inversely with temperature (figure 7*a*); the dependence was particularly strong for samples with *x* ≤ 0.25 as a result of Umklapp scattering due to phonon–phonon interactions at elevated temperatures [47]. With increasing Pr content, the dependency reduced, such that it was almost temperature independent for samples with *x* = 1.0. For example, *κ*_total_ varied between 6.5 and 4 W m^−1^ K^−1^ for samples with *x* = 0.10, while it was almost constant at approximately 2 W m^−1^ K^−1^ for samples of *x* = 1.0. This temperature independence of *κ*_total_ has also been reported for other A-site deficient perovskites such as, La_1/3_NbO_3_ [48], Nd_2/3_TiO_3_ [49,50] and Sr_1−x_La_2x/3_□_x/3_TiO_3_ [12,14]. As the microstructural features in the present investigation (figure 2) are all much larger than the phonon mean-free path (*l_ph_*) for SrTiO_3_ (less than 10 nm) [7,51], the temperature dependence of *κ*_total_ cannot be linked to simple bulk microstructural effects, but can be explained by changes in crystal structure and specifically changes in lattice thermal conductivity (*κ*_lattice_). By using the Wiedemann–Franz law to calculate electronic thermal conductivity (*κ*_electronic_ = L*σ*T; *L* = 2.44 × 10^−8^ W Ω K^−2^), it was possible to calculate lattice thermal conductivity (*κ*_lattice_ = *κ*_total_ – *κ*_electronic_). Data at 373 and 973 K for the full range of samples are presented in figure 7*b*. To provide insight into the thermal transport processes occurring in Sr_1−x_Pr_2x/3_□_x/3_TiO_3_, *l_ph_* values for the ST–PT samples were calculated using the method of Popuri *et al.* [12], where sound velocity is estimated from the Debye temperature (*θ*_D_ = 513 K) [52] and then *l_ph_* is extracted through its relationship with *κ*_lattice_. It was found that *l_ph_* decreases from 18.3 to 7.2 Å for 0.10 ≤ *x* ≤ 1.0, supporting the existence of a glassy state since *l_ph_* was comparable to that of interatomic distances [12]. Moreover, the *l_ph_* values for *x* ≥ 0.70 compositions were similar to the *l_ph_* of crystalline Quartz glass (≈7.8 Å at room temperature [53]). Therefore, the introduction of a mass contrast (substituting Sr for the heavier Pr) led to the formation of a layered crystal structure upon Pr/vacancy doping; this, in addition to the non-uniform distortion of TiO_6_ octahedra, and the presence of anti-phase and twin boundaries, is responsible for the effective creation of a glassy state in the crystalline material [14,54,55]. Finally, the variation in *κ*_lattice_ did not follow that of oxygen deficiency (deduced from TGA), suggesting that the Pr/vacancy concentration had a more dramatic effect on thermal transport than oxygen vacancies.

The temperature dependence of *ZT* with respect to composition is presented in figure 7*c*. *ZT* values varied between 0.03 and 0.23 across the full range of measurement temperature (323–867 K) with a maximum *ZT* of approximately 0.23 at 867 K for samples of *x* = 0.25. However, a further increase in *x* resulted in the reduction of *ZT*. Interestingly, *ZT* was not significantly affected by Pr/vacancy concentration for *x* ≥ 0.4 (figure 7*d*). For the Pr end member in the solid solution series, Pr_2/3_TiO_3±*δ*_, a maximum *ZT* of 0.16 at 865 K was achieved, which is the first report of the thermoelectric properties for this material. For the full system, the *ZT* values are comparable with the findings of Azough *et al.* [14] for Sr_1−_*_x_*La_2*x*/3_□*_x_*_/3_TiO_3_. However, *ZT* values as high as 0.41 have been reported for La-doped SrTiO_3_ [13].

### (f) Effect of carbon burial sintering

In order to evaluate the potential benefit to properties of a sintering atmosphere provided by carbon burial [56], the same types of samples were encased in graphite for sintering. The samples processed under graphite achieved notably higher density (greater than 96% theoretical) than samples sintered in H_2_–Ar atmosphere, in spite of the much shorter processing time. Carbon burial sintering is therefore an effective route for the production of high-quality samples.

XRD analysis (electronic supplementary material, figure S3) showed that the changes in crystal structure with Pr/vacancy concentration were similar to those of samples sintered in H_2_–Ar atmosphere; (i) cubic for *x* ≤ 0.10; (ii) tetragonal for 0.10 < *x* ≤ 0.85 and (iii) orthorhombic for *x *< 0.85. However, the composition at which long-range cation/vacancy ordering was detected increased to *x *> 0.85 and the presence of a Magnéli phase was more apparent. This suggests that the amount of the secondary phase increased in samples sintered in graphite and that the encasing procedure provides a more aggressive reducing environment for the samples [24]. This is supported by the lattice parameter data for *a*_*pc*_ (electronic supplementary material, figure S4), which were larger than for samples sintered in an H_2_–Ar atmosphere, indicating a higher concentration of oxygen vacancies and [Ti^3+^] [10].

**Figure 8.**
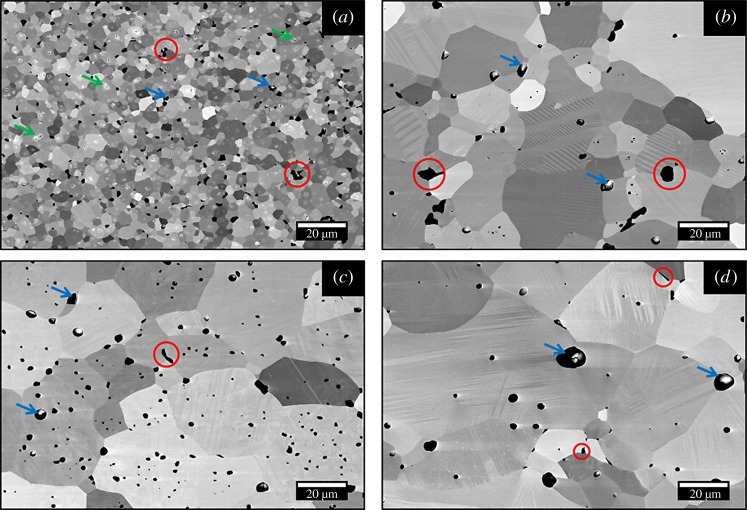
SEM-BSE micrographs for the samples prepared in graphite; (*a*) X10-CM, (*b*) X40-CM, (*c*) X70-CM and (*d*) X100-CM samples. (Online version in colour.)

SEM microstructures for the samples prepared under graphite varied with Pr/vacancy concentration (figure 8); core-shell type structures were observed for *x* ≤ 0.25 (see electronic supplementary material, figure S5) while the compositions with higher *x* exhibited large grains with phase transformation-induced domain features [14]. The average grain size changed from approximately 3–44 µm with increasing x for 0.10 ≤ *x* ≤ 1.0. The grain sizes for samples of higher Pr content were significantly larger than for the equivalent samples sintered under H_2_–Ar atmosphere.

**Figure 9.**
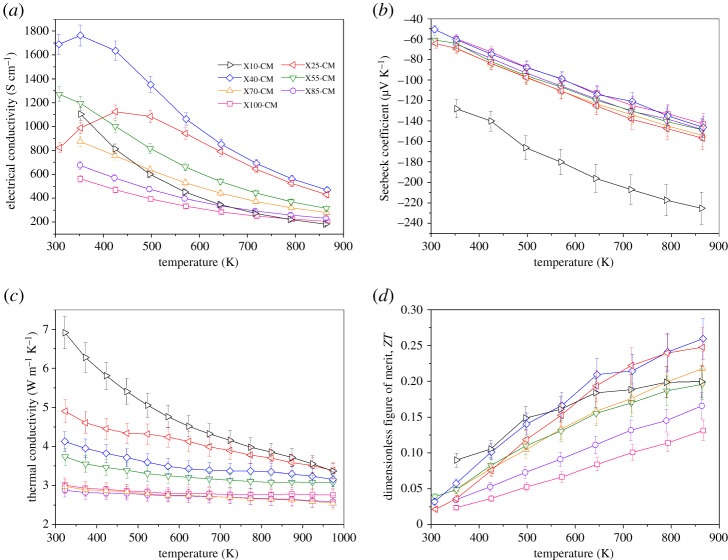
Temperature dependencies of (*a*) electrical conductivity, (*b*) Seebeck coefficient, (*c*) total thermal conductivity and (*d*) *ZT* values of the samples prepared in graphite. (Online version in colour.)

The electrical conductivity of the samples sintered in graphite (figure 9*a*) followed the same compositional dependence as samples processed in H_2_–Ar (figure 6*a*) but the former was greatly enhanced by the increased carrier concentration (see electronic supplementary material, figure S6—data from, XPS and TGA analysis). For example, the electrical conductivity of samples of *x* = 0.1 increased from approximately 340 S cm^−1^ to approximately 1100 S cm^−1^. The increase in carrier concentration led, as expected, to a reduction in the Seebeck coefficients (figure 9*b*); for example, for X10-CM (carbon bed samples) values were between −125 and −225 µV K^−1^, while for X10-HA (H_2_–Ar atmosphere) values were higher at −140 to −250 µV K^−1^. The net benefit of the graphite processing environment becomes apparent in the data for maximum power factor (electronic supplementary material, figure S7) increasing, for example, to approximately 1.80 × 10^−3^ W m^−1^ K^−2^ at 350 K for *x* = 0.10. This type of increase is reflected in all other samples sintered under graphite, with an average *σ*S^2^ value of 1.0 × 10^−3^ W m^−1^ K^−2^ for compositions with *x* ≤ 0.40 from 475 to 975 K. This improvement was due to the significant enhancement of *σ* dominating the reduction of |S| with carbon burial sintering, together giving some of the highest values reported for SrTiO_3_-based thermoelectrics [16].

The temperature dependence of total thermal conductivity (*κ*_total_) for the samples sintered in graphite (figure 9*c*) is very similar to that for samples produced under a gas atmosphere. Thermal conductivity of X10-CM samples (graphite bed) varied between 6.9 and 3.44 W m^−1^ K^−1^, while for X100-CM samples *κ*_tota_ was stable at approximately 2.8 W m^−1^ K^−1^. This shows the increased contribution of electrons to thermal transport after sintering in graphite as a result of increased carrier concentration. Finally, the *ZT* values for the samples prepared under graphite (figure 9*d*) confirm the benefit of the alternative processing strategy. In general, *ZT* values were increased; the composition at which the maximum *ZT* occurred changed to *x* = 0.40, with *ZT* of 0.26 at 865 K. Moreover, for samples of *x* = 0.25 the maximum *ZT* increased slightly to 0.25 at 865 K. However, for samples of *x* = 1.0, the maximum *ZT* decreased to 0.13 at 865 K as a result of a notable increase in *κ*_total_. Collectively, the results suggest that sample preparation in graphite maintains or increases the *ZT* values while enhancing the power factor. Therefore, the approach provides a route to process thermoelectric oxides which enables maintained/improved conversion efficiency while enhancing output power.

The Sr_1−*x*_Pr_2*x*/3_□*_x_*_/3_TiO_3_ compositions presented here exhibit at least similar or superior properties to the earlier findings on Sr_1−*x*_La_2*x*/3_□*_x_*_/3_TiO_3_ [14]. Preparation of the two types of samples in H_2_–Ar atmosphere yields similar electrical conductivity values, while sintering in a graphite bed led to an enhancement in the electrical conductivity, leading to a notable increase in the power factor of Sr_1−*x*_Pr_2*x*/3_□*_x_*_/3_TiO_3_ over that of Sr_1−*x*_La_2*x*/3_□*_x_*_/3_TiO_3_ by 9% for sintering in H_2_–Ar, and by 14% for sintering graphite. Interestingly, in comparison to their La-doped counterparts, the samples prepared in this study exhibited higher thermal conductivity at low temperatures and lower thermal conductivity at higher temperatures. Such behaviour is consistent with the larger electronic thermal conductivity contribution at low temperatures while the effect of the heavier dopant (Pr) begins to dominate at high temperatures. Therefore, similar *ZT* values were achieved for Sr_1−*x*_Pr_2*x*/3_□*_x_*_/3_TiO_3_ and Sr_1−*x*_La_2*x*/3_□*_x_*_/3_TiO_3_; for example the maximum *ZT* for Sr_0.5_La_0.33_TiO_3_ was 0.27 at 870 K and for Sr_0.6_Pr_0.267_TiO_3_ 0.26 at 866 K, indicating that maximum *ZT* was not dependent on the lanthanide dopant, because of the simultaneous, contrasting modification of electronic and thermal transport properties of the Pr and La analogues.

## 4. Conclusion

High quality, dense (1 − *x*) SrTiO_3_ − (*x*) Pr_2/3_ □ _1/3_TiO_3_ ceramics were prepared for the first time by two different approaches; directly sintering in H_2_–Ar atmosphere and carbon burial sintering. By varying the Pr/vacancy concentration three different crystal structures developed, irrespective of processing route; cubic (*a*^0^*a*^0^*a*^0^), tetragonal (*a*^0^*a*^0^*c^–^*) and orthorhombic (*a^–^a^–^c^+^*) structures for low, medium and high levels of doping, respectively. The lattice parameter, *a*_pc_ decreased with increasing doping concentration.

HRTEM-SAED showed that samples of *x* = 1.0 had a layered structure with alternating fully and partially occupied A-sites and a short-range order along the (100) direction, suggesting a variation in the occupancy of the partially occupied layers.

Features in the microstructure depended on composition. For *x *< 0.25 there are grains with core-shell features, and for *x *> 0.25 phase transformation-induced domain structures; samples of *x* = 0.25 exhibit a combination of both. Average grain size increased with increasing Pr/vacancy concentration.

The electrical conductivity is sensitive to the changes in crystal structure with composition. High electrical conductivity is associated with the high symmetry structure at low values of *x*. By contrast, the lower symmetry structures at higher values of *x*, where there is increased distortion of the Ti–O octahedra, leads to a reduction of the electrical conductivity. The maximum electrical conductivity occurred in samples of *x* = 0.25, and this enabled a very high power factor of approximately 1.75 × 10^−3^ W m^−1^ K^−2^ at 425 K to be achieved. The existence of a relatively high and stable power factor over the wide operating temperature range (425–875 K) for *x* ≤ 0.40 makes these materials attractive for thermoelectric applications.

Thermal conductivity decreased with increasing Pr/vacancy doping but is also sensitive to crystal structure and the presence of sub-grain features. The large mass contrast and strain field induced by Pr/vacancy doping, the formation of long-range cation–vacancy ordering and the presence of non-uniform Pr/vacancy distributions in the ordered structure encouraged phonon scattering; the phonon mean-free path, *l_ph_*, is comparable to interatomic distances. As a consequence, the samples with *x* ≥ 0.85 exhibited glass-like lattice thermal conductivity.

Carbon burial sintering is an effective and robust method for the preparation of SrTiO_3_-based ceramics. It does not significantly alter the crystal structure or microstructure but increases the oxygen deficiency leading to an increase in the carrier concentration. The maximum power factor of approximately 1.80 × 10^−3^ W m^−1^ K^−2^ was achieved for *x* = 0.10 at 350 K after sintering in graphite; one of the highest reported for SrTiO_3_-based ceramics. The maximum *ZT* was marginally increased upon sintering in graphite (e.g. from *ZT* = 0.25 for *x* = 0.25 at 865 K) to *ZT* of 0.26 for samples of *x* = 0.40 at 865 K when sintered in graphite. The synthesis strategies employed here can be developed and exploited in a much wider range of materials to control and enhance functional performance.

## Supplementary Material

TGA, XRD, XPS and thermoelectric data

## Data accessibility

This article has no additional data.
