# Supplementary material for: Enhancing the thermoelectric properties of Sr1−xPr2x/3□x/3TiO3±δ through control of crystal structure and microstructure
Source: Philos Trans A Math Phys Eng Sci. 2019 Jul 8;377(2152):20190037. doi: 10.1098/rsta.2019.0037 (PMC6635635; doi:10.1098/rsta.2019.0037)
Supplement: TGA, XRD, XPS and thermoelectric data [file rsta20190037supp1.docx]

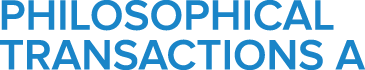
 Phil. Trans. R. Soc. A.

doi:10.1098/not yet assigned

**Enhancing the Thermoelectric Properties of** Sr_1−x_Pr_2x/3_□_x/3_TiO_3_**_±δ_** **through Control of Crystal Structure and Microstructure**

Dursun Ekren, Feridoon Azough, Robert Freer*

*School of Materials, University of Manchester, Manchester M13 9PL, U.K*.

^*^corresponding author: [Robert.Freer@manchester.ac.uk](mailto:Robert.Freer@manchester.ac.uk); [0000-0003-1100-8975](http://orcid.org/0000-0003-1100-8975)

## Supplementary Information


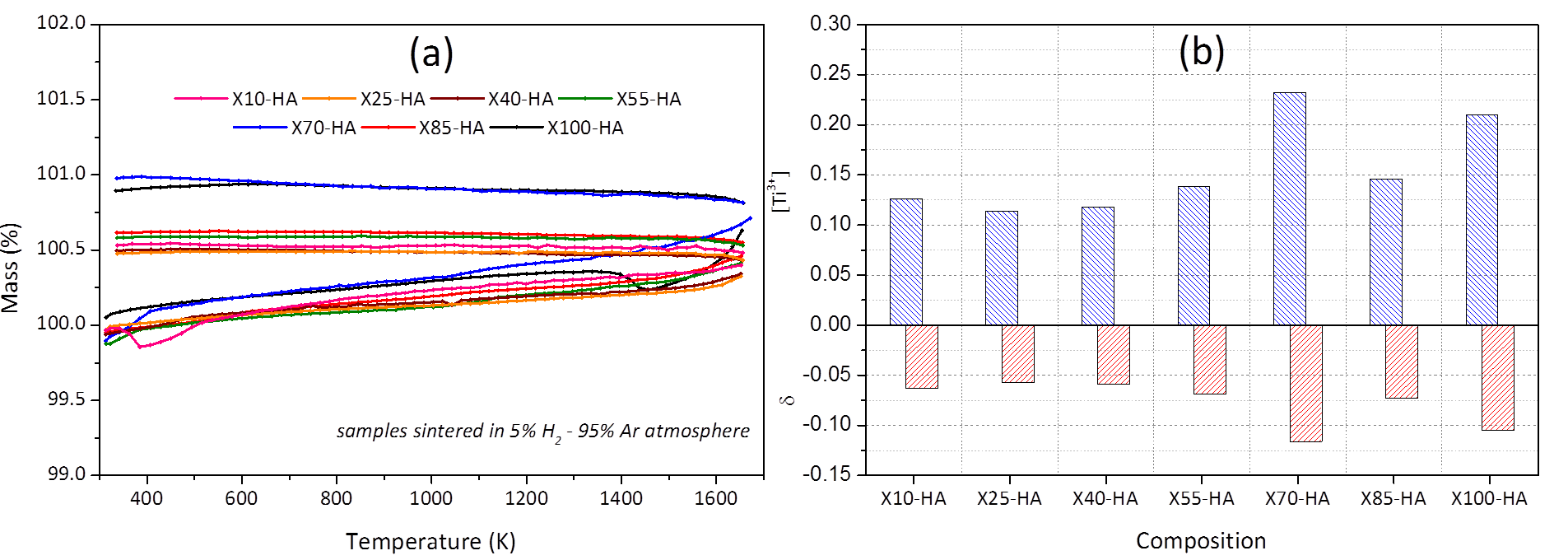


**Figure S1** **(a)** TGA curves and **(b)** [Ti^3+^] and δ values obtained from the TGA experiments for the samples sintered H_2_-Ar atmosphere

*
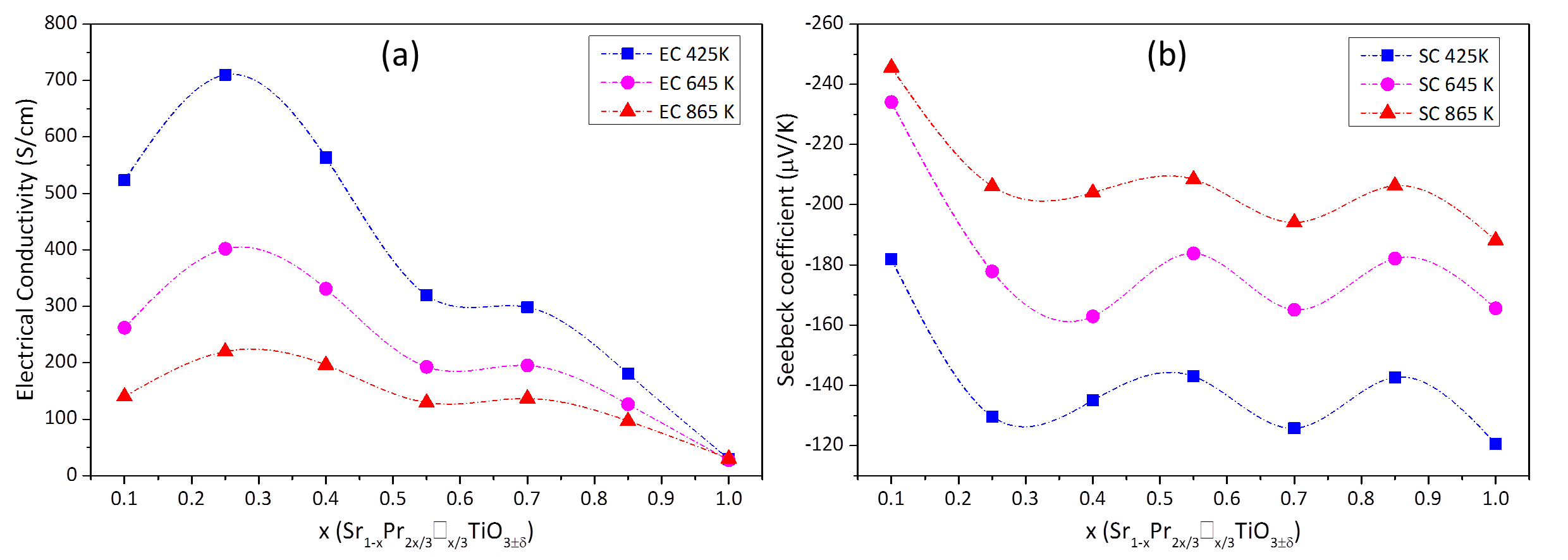
*

**Figure S2** **(a)** Electrical conductivity and **(b)** the Seebeck coefficient of Sr_1−x_Pr_2x/3__x/3_TiO_3±δ_ compositions at different temperatures (425, 645 and 865 K) with respect to x.


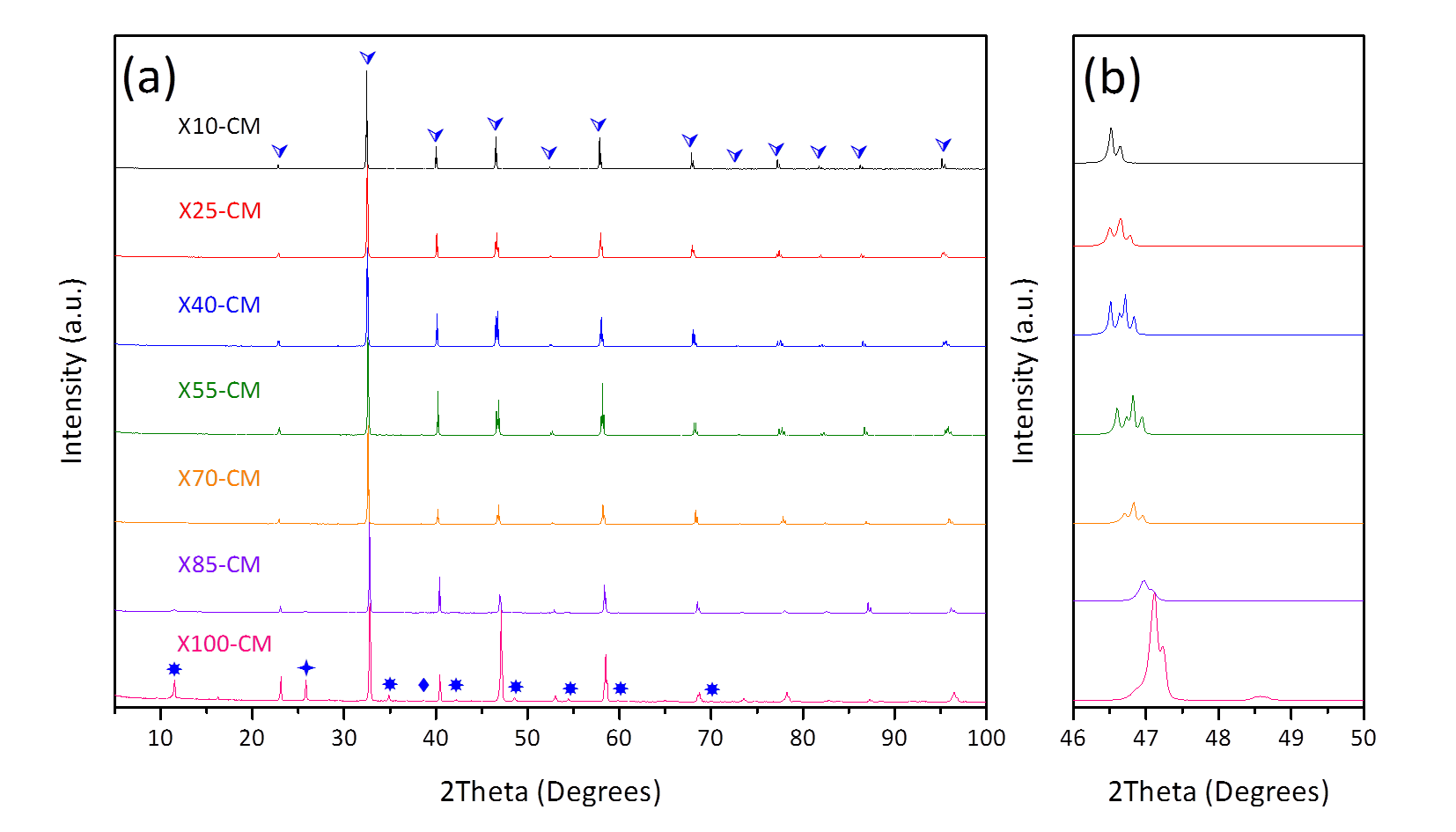


**Figure S3** **(a)** XRD spectra and **(b)** (200)_cubic_ peak region for ST-PT samples sintered in graphite. In the figure, ⮛, ⯁, 🟄 and ✹ correspond to *Pm*$\bar{3}$*m* SrTiO_3_, R-point reflections, M-point reflections and X-point reflections respectively.


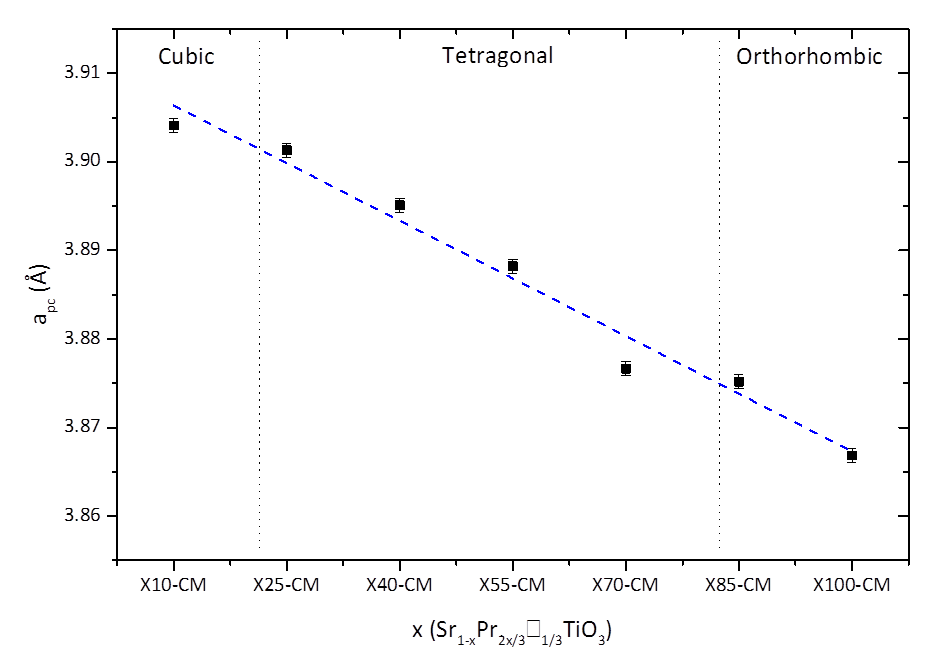


**Figure S4** Effect of composition on the lattice parameter, *a_pc_* for ST-PT samples sintered in graphite

*
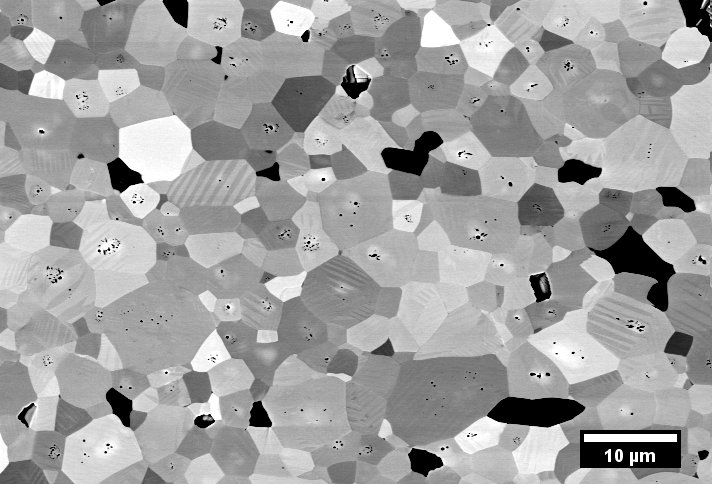
*

**Figure S5** SEM micrograph of Sr_0.75_Pr_0.167_TiO_3±δ_ (x = 0.25) showing domain features in addition to the core-shell type structures in grains


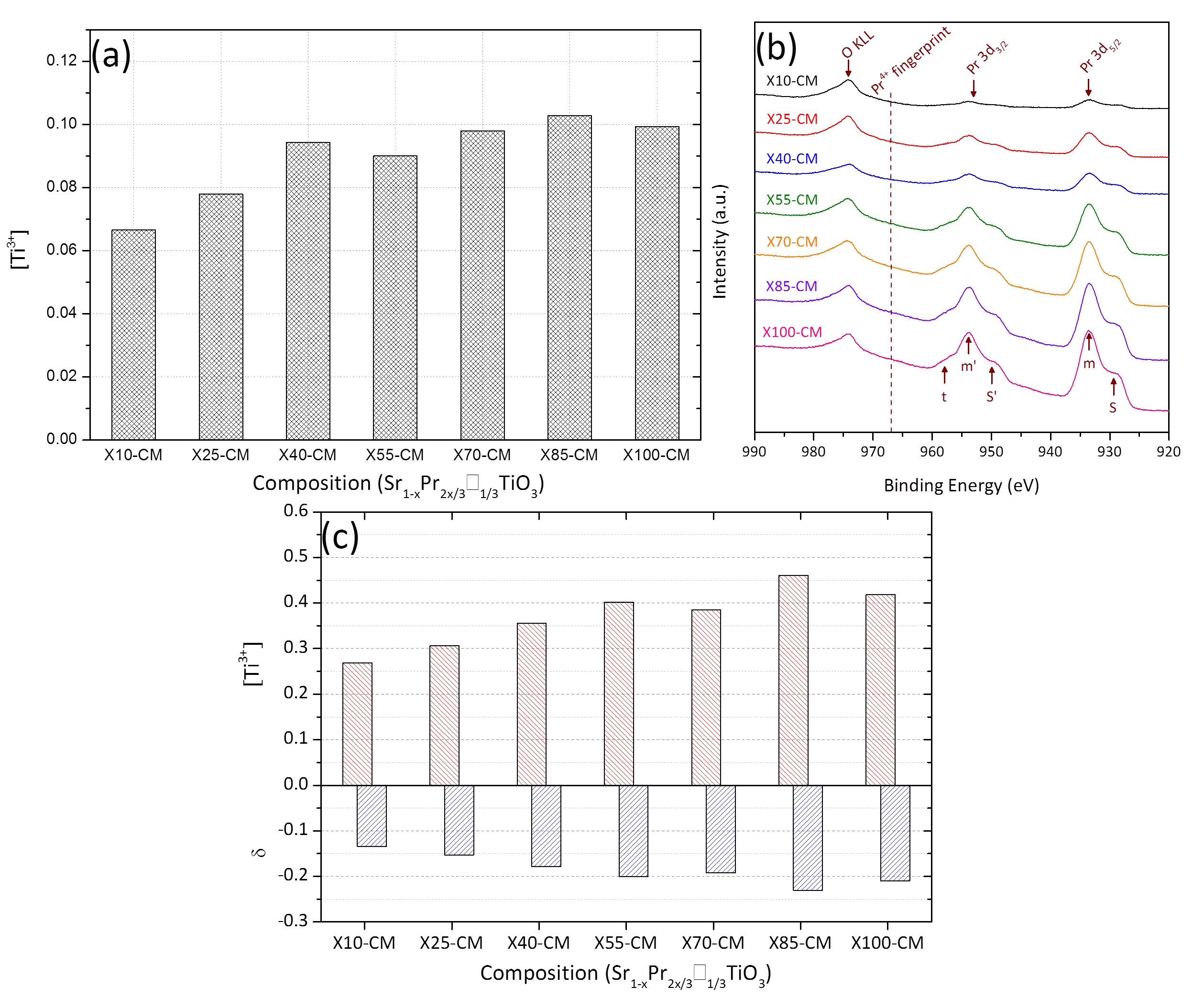


**Figure S6** **(a)** [Ti^3+^] from fitting process of XPS data collected, **(b)** High resolution Pr 3d spectra with respect to composition and (c) [Ti^3+^] and δ values obtained from TGA results for the samples sintered in graphite


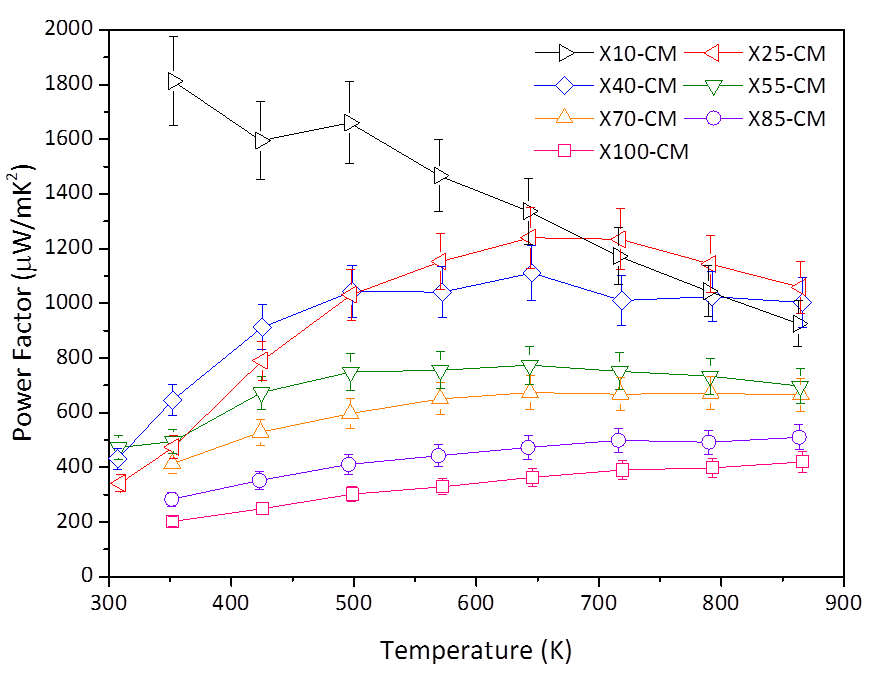


**Figure S7** Temperature dependence of the thermoelectric power factor of ST-PT samples sintered in graphite
